# Real-Time Intracellular Monitoring of miRNA Dynamics during Induced Pluripotent Stem Cell Neuronal Differentiation via Plasmon-Enhanced Nanobiosensing

**DOI:** 10.1021/acs.nanolett.5c01840

**Published:** 2025-06-10

**Authors:** Yannan Hou, Meizi Chen, Letao Yang, Ki-Bum Lee

**Affiliations:** † Department of Chemistry and Chemical Biology, 242612 Rutgers, The State University of New Jersey, Piscataway, New Jersey 08854, United States; ‡ Shanghai Tongji Hospital, Key Laboratory of Spine and Spinal Cord Injury Repair and Regeneration, Ministry of Education, Frontier Science Center for Stem Cell Research, School of Life Sciences and Technology, Tongji University, Shanghai 200092, China

**Keywords:** Intracellular miRNA Detection, Real-time Monitoring, Gold Nanorods, Nondestructive Biosensor, Monitoring iPSC Differentiation, Metal-Enhanced Fluorescence (MEF), Neuronal Differentiation

## Abstract

Induced pluripotent stem cells (iPSCs) offer immense potential for treating central nervous system (CNS) disorders and injuries. However, the lack of highly sensitive, selective, and noninvasive biosensors for real-time monitoring of iPSC neuronal differentiation remains a critical barrier. In this work, we introduce a gold nanorod-based metal-enhanced molecular beacon (MEMB) nanobiosensor for the noninvasive, real-time detection of intracellular miRNA-124, a key biomarker for neuronal differentiation in human iPSC-derived neural stem cells. Designed through finite-difference time-domain (FDTD) simulations and experimentally validated for optimized localized surface plasmon resonance (LSPR) properties, MEMB nanobiosensors achieved picomolar-level sensitivity and single-mismatch selectivity toward miRNA-124 detection, along with great biocompatibility demonstrated by live-cell assays. Collectively, the MEMB platform provides a robust analytical tool for in-depth investigations of molecular and genetic regulatory networks during iPSC neuronal differentiation in a nondestructive manner, paving the way toward safer, more efficient, and better-characterized iPSC-derived cell therapies for CNS diseases and injuries.

## Graphical abstract

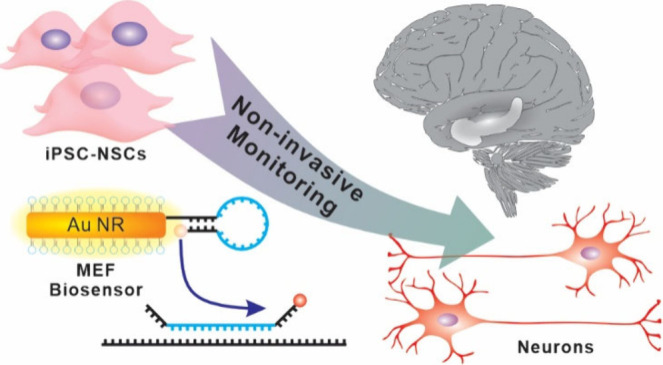

Central nervous system (CNS) disorders, encompassing neurodegenerative diseases (such as Huntington’s, Parkinson’s, and Alzheimer’s diseases) and traumatic injuries (affecting the spinal cord and brain), collectively impact millions of patients worldwide.^1–4^ These conditions frequently result in debilitating outcomes and present significant therapeutic challenges, with many lacking definitive curative interventions.^5^ To address these challenges, patient-derived induced pluripotent stem cells (iPSCs) and their neural stem cell derivatives (iPSC-NSCs) have emerged as promising platforms for investigating neurological disorders through disease-specific models (e.g., iPSC-derived disease models^6–12^ ) and for developing therapeutic interventions (e.g., iPSC-based stem cell therapy^13–16^ ) via stem cell-based approaches, offering several key advantages.^17–20^ First, iPSC-derived disease models retain human genetic backgrounds that are difficult to realize using animal models.^21,22^ Second, iPSCs can be generated from a patient’s own cells and subsequently transplanted back into the same patient (autologous transplantation), which significantly minimizes the risk of immune rejection.^23^ Despite the bright future, bench-to-bedside translation of iPSCs has been strongly restricted, partially due to a lack of accurate and reliable methods to characterize iPSCs.^24^ During reprogramming and differentiation, iPSCs undergo extensive remodeling of their molecular and genetic regulatory networks.^25^ Various noncoding RNAs (ncRNAs), including microRNAs (miRNAs), PIWI-interacting RNAs (piRNAs), enhancer RNAs (eRNAs),^26^ and circular RNAs (circRNAs),^27,28^ function as critical regulatory elements and serve as distinctive molecular signatures of iPSC neurogenesis. For example, miRNA-124 has been established as a hallmark of neuronal differentiation,^29^ while the upregulation of let-7 miRNA implies Müller glial cell reprogramming.^30^ Despite their critical roles, monitoring the dynamic and spatial distribution of ncRNAs within iPSCs remains technically challenging. Overcoming this challenge by developing methods to accurately track these ncRNAs would address significant knowledge gaps in understanding the molecular and genetic regulatory mechanisms governing iPSC fate determination. Such advancements could ultimately inspire novel strategies to enhance both the efficacy and safety of iPSC-based therapeutic applications.

To study the dynamics of ncRNAs during neural differentiation of iPSCs and adult stem cells, conventional approaches, such as quantitative polymerase chain reaction (qPCR),^31,32^ RNAscope,^33^ and fluorescence in situ hybridization (FISH),^34^ have been widely applied. However, these approaches typically require the sacrifice of stem cells before the analysis can be performed. Genetically encoded biosensors (e.g., GFP-tag targeting specific proteins^35^) can monitor distinct proteins in living cells.^36^ Still, they would require gene-editing of the iPSCs prior to differentiation, which becomes complicated during clinical translation.^37^ Our group and others have developed various biosensor platforms, including electrochemical,^38^ magnetic,^39,40^ and optical systems^41–44^ capable of monitoring stem cell differentiation by detecting extracellular transmitters or biomolecules in stem cell-derived exosomes. However, these approaches generally fail to provide high-resolution spatial information regarding the intracellular distribution of these biomolecules.^45,46^

The rise of molecular beacon-based biosensors has provided a unique solution for real-time and multiplex detection of biomarkers, including miRNAs.^47–50^ Nevertheless, fluorescence-based detection methods frequently suffer from low signal-to-noise ratios and insufficient detection sensitivity due to cellular autofluorescence, which limits their ability to accurately quantify biomolecules present at sub-nanomolar concentrations.^51^ This raises significant concerns, as the majority of ncRNAs are present at sub-nanomolar levels^52^ Although plasmonic nanoparticle-based Raman biosensors have been demonstrated with ultrasensitive detection of noncoding RNAs or proteins, their signal instability and relatively slow detection speed strongly restrict their applications in monitoring iPSC differentiation.^53^ Therefore, current methods remain largely inadequate for the sensitive, selective, and real-time monitoring of the spatiotemporal distribution of RNA during iPSC differentiation.

To overcome these challenges, we developed a novel nanobiosensing platform for highly sensitive, selective, and reliable noninvasive monitoring of intracellular ncRNAs. This platform enables us to study the neuronal differentiation of human patient-derived iPSC-derived neural stem cells (hiPSC-NSCs) [Figure 1]. The nanobiosensor is built upon molecular beacon (MB)-assembled gold nanorods with optimal localized surface plasmonic resonance (LSPR) for metal-enhanced fluorescence (MEF) [Figure 1A]. By building a library of nanorods and identifying the most optimal structure for MEF, the fluorescence of dye-labeled MB could be exponentially amplified when bound to the target RNA or DNA sequences, thereby enhancing the detection limit while maintaining a high detection specificity and signal stability for the MB [Figure 1B]. Notably, the metal-enhanced molecular beacon (MEMB) has a size (15 nm by 45 nm) optimal for endocytosis and cellular internalization,^54^ thereby circumventing the challenge of delivering negatively charged nanomaterials through cellular membranes and making the system ideal for real-time monitoring of intracellular biomolecules [Figure 1C]. To demonstrate this concept, we engineered a MEMB nanobiosensor specifically designed to detect miRNA-124, a well-established neurogenic regulatory ncRNA whose expression levels strongly correlate with the differentiation state of neural stem cells [Figure 1D]. Remarkably, the MEMB nanobiosensor exhibits exceptional sensitivity, achieving a picomolar detection limit for miRNA-124, and demonstrates high selectivity by effectively discriminating between sequences with single-nucleotide mismatches. In solution-based assays, the MEMB also maintains signal-to-noise ratios exceeding 10, thus overcoming challenges with conventional approaches. Furthermore, MEMB readily internalizes into iPSC-NSCs, enabling real-time, noninvasive monitoring of dynamic fluctuations in intracellular miRNA-124 levels throughout the neuronal differentiation process [Figure 1E]. In short, these capabilities establish MEMB as a powerful analytical tool for investigating the intricate molecular and genetic regulatory networks governing neurogenesis, aiding to accelerate the development of safe and effective iPSC-based therapeutic approaches.

**Figure 1:**
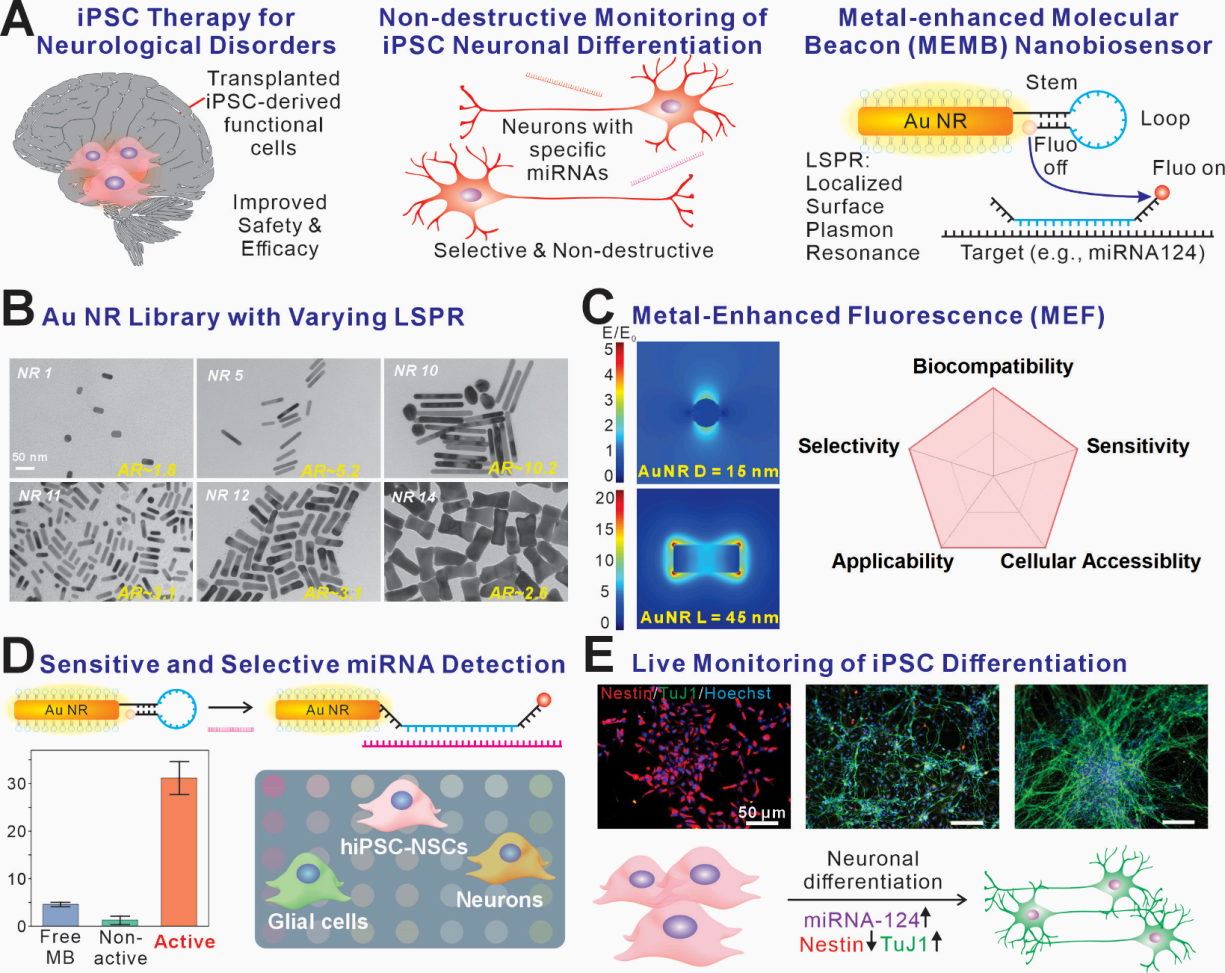
MEMB monitoring neurogenic differentiation by the selective detection of miRNA-124. A) Schematic diagram of the mechanism of the MEMB nanobiosensor monitoring iPSC neuronal differentiation. Molecular beacons conjugated with AuNRs with optimal LSPR for MEF upon the hybridization of the MB loop with the target miRNA. B) TEM images showing the comprehensive library of AuNRs with various sizes and aspect ratios. C) FDTD simulation of electromagnetic field enhancement, suggesting strong MEF on the surface of AuNRs (left), ensured the high sensitivity of MEMB miRNA detection. D) Selective detection of the target sequence ensured cell-type-specific monitoring of iPSC differentiation. E) Nondestructive live monitoring of iPSC neuronal differentiation by intracellular monitoring of miRNA-124 aligned with the immunocytochemistry analysis result of neuronal marker TuJ1 (green) and stem cell marker Nestin (red). Scale bar = 50 μm.

The MEMB nanobiosensor achieves exceptional sensitivity in miRNA detection through the LSPR-mediated MEF effect.^55^ To optimize this LSPR-induced fluorescence enhancement, we systematically developed a comprehensive library of gold nanorods with precisely controlled aspect ratios and dimensions, yielding distinct plasmonic properties that serve as optimal building blocks for the MEMB platform. Plasmonic gold nanostructures, with their unique optical and electronic properties, have been extensively utilized in various biosensing applications, providing significant advantages in terms of detection sensitivity and specificity. The LSPR phenomenon originates from the coherent oscillation of the electron cloud under the external electromagnetic field, which leads to a nonlinearly amplified electromagnetic field that enhances the absorption, scattering, or fluorescence on the surface of noble metal structures.^56^ The amplification is highly dependent on the dielectric coefficient, size, shape of the plasmonic nanostructures, and the wavelength and strength of the external electromagnetic field.^57,58^ Although noble metals such as silver can support strong LSPR, they were limited in biosensing applications due to their low chemical stability in aqueous and biological environments, such as their tendency to oxidize and release toxic silver ions that can cause cytotoxicity and harm biological systems.^59–61^ Although a larger nanorod (with a longer major axis) may allow more beacons to bind to the surface, it may not provide as efficient cellular uptake. Therefore, we focused on gold nanostructures due to their higher biocompatibility, stability, and ease of surface functionalization. Although gold microstructures such as nanoparticle aggregates and microrods can also induce stronger LSPR with hotspots located between adjacent nanostructures,^62^ we decided to focus on smaller (<100 nm) gold nanorods for efficient cellular uptake and better signal stability.

We adapted the well-established seed-mediated method for the synthesis of gold nanorods.^63–65^ The redox reaction between chloroauric acid and sodium borohydride in the presence of a capping reagent of cetrimonium bromide (CTAB) yielded gold nanoclusters as seeds to direct the growth into one-dimensional (1D) nanorod crystals. To initiate the anisotropic crystal growth, silver nitrate was then mixed with the gold nanoclusters and capping agent CTAB, followed by the reduction of a mild reducing agent, ascorbic acid [Figure 2A]. Based on this mechanism, we could effectively modulate the size, shape, and aspect ratio of gold nanorods by varying the ratio between chloroauric acid and silver nitrate, the concentration of capping agent CTAB, pH, and reducing agents.^65^ By carefully tuning these experimental parameters, we built a comprehensive gold nanorod library with precise control over aspect ratios of nanorods ranging from 1 to 10 [Figure 2B], the minor axis of the nanorods spanning from 10 to 80 nm, and the long axis of the nanorods spanning from over 10 nm to around 100 nm [Figure S1]. UV–vis spectra of synthesized gold nanorods showed the characteristic 520 nm peak corresponding to the transverse electronic oscillation, independent of nanorod sizes, as well as the longitudinal electronic oscillation peak, whose wavelength is proportional to the aspect ratio of nanorods [Figure S2]. Considering that CTAB could be cytotoxic to iPSCs, we next went on to remove CTAB from the gold nanorods. However, the immediate removal of CTAB led to irreversible nanorod aggregation, which compromised their functionality for intracellular miRNA detection. Therefore, we first stabilized the gold nanorod by noncovalent functionalization with anionic poly­(sodium 4-styrenesulfonate) (PSS), then performed a ligand exchange with biocompatible citrate as the capping agent.^66^ The resulting nanorods demonstrate excellent colloidal stability and facilitate further covalent conjugation with thiol-terminated molecular beacons. Specifically, we tested the biocompatibility of citrated capped nanorods in human iPSC-derived NSC using the standard PrestoBlue assay [Figure S6]. Based on our cytotoxicity assays, we determined that citrate-capped gold nanorods did not exhibit significant cytotoxicity at concentrations up to 100 μg/mL, which is well above the concentration range typically employed for cellular monitoring experiments. These results convincingly demonstrate the excellent biocompatibility of our synthesized gold nanorods, validating their suitability and safety for the development of intracellular nanobiosensors targeting the real-time monitoring of biomolecular dynamics within iPSC-derived cells.

**Figure 2:**
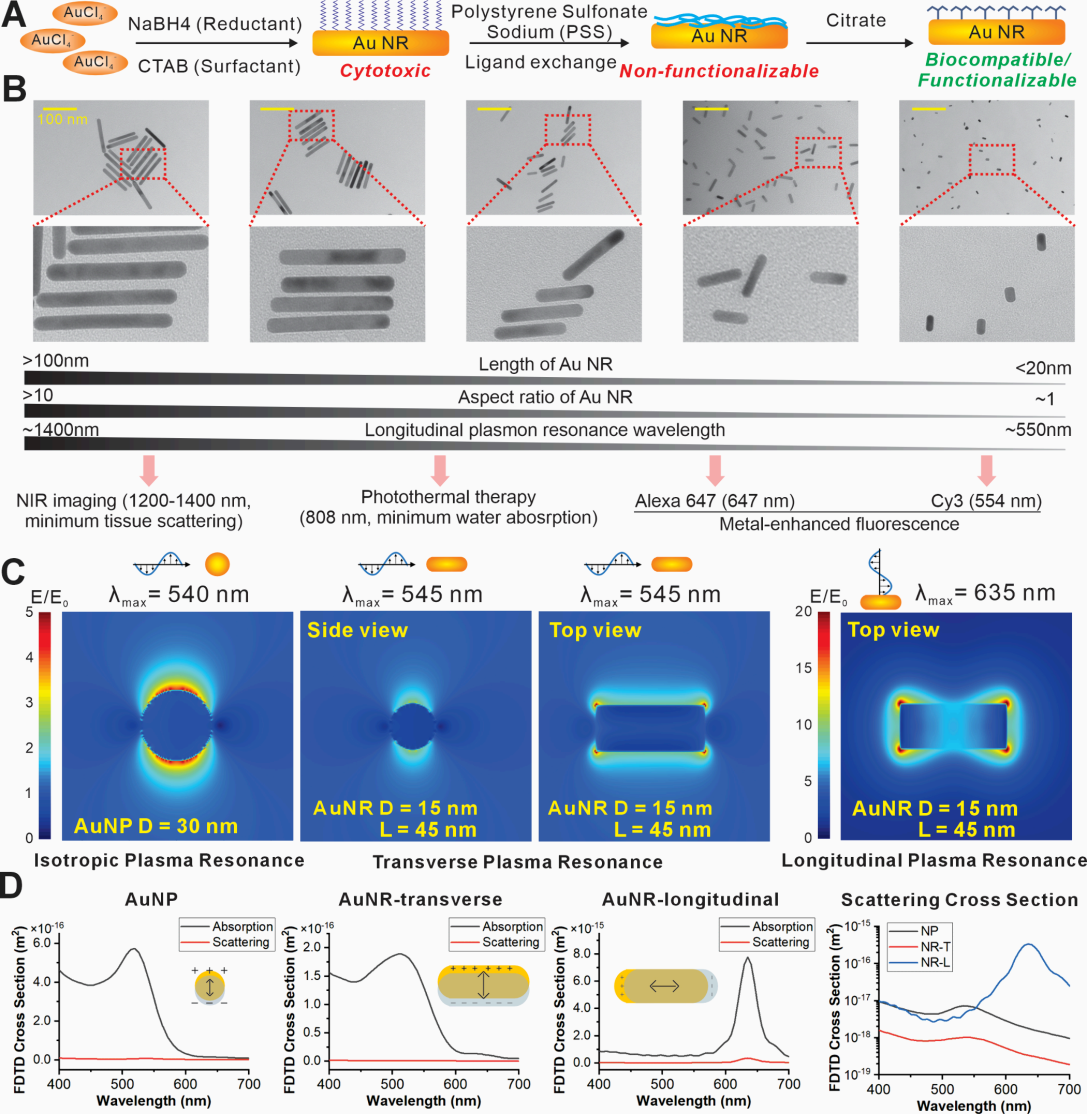
Gold nanorod synthesis and FDTD simulation of AuNR LSPR. A) Bottom-up synthesis of AuNR and surface modification. AuNRs were synthesized through a seed-mediated wet chemistry method. Gold seeds were formed by reducing chloroauric acid with sodium borohydride, with CTAB as a capping reagent. Anisotropic growth of gold nanorods was carried out in the presence of silver nitrate, CTAB, and ascorbic acid. Purified AuNRs capped by CTAB went through a two-step surface ligand exchange process with PSS and sodium citrate, respectively, to improve the biocompatibility and surface modification ability of AuNRs. B) TEM images characterizing synthesized AuNRs with controlled sizes, aspect ratios, and LSPR wavelengths. C) FDTD simulation of electromagnetic field enhancement at the surface of AuNRs (15 nm by 45 nm) and 30 nm gold nanoparticle (AuNP). AuNR demonstrated the most substantial electromagnetic field enhancement (longitudinal mode) when its major axis is parallel with the polarization plane of the excitation wave. D) FDTD simulation of absorption and scattering cross sections of AuNR and AuNP. The scattering cross-section area was calculated to be the largest for AuNRs in their longitudinal mode at an excitation wavelength of around 635 nm.

Next, we investigated the MEF effect across our diverse gold nanorod structures through computational optical simulations. The LSPR properties of plasmonic nanostructures are heavily dependent on their geometric parameters (size and shape), as well as on the characteristics of the incident electromagnetic field (wavelength, polarization, and intensity).^67^ To comprehensively explore and optimize these parameters, we employed finite-difference time-domain (FDTD) simulations to systematically model the electromagnetic interactions between incident light and our library of gold nanorods with varying dimensions. We selected Alexa Fluor 647 as the fluorescent label for the molecular beacon, as it is located in the near-infrared (NIR) range and shows relatively low absorption by water, cell media, or biological fluids.^68,69^ Therefore, it would be ideal to tailor the nanorod structures with an LSPR peak close to 647 nm to maximize the MEF. First, we applied FDTD calculation to model the light-matter interactions between the laser and the gold nanorods with varying sizes and shapes that we synthesized in the library. Given that the dimensions of nanorods are typically between 10 and 80 nm, a small domain feature of 0.5 nm was used in the calculation to achieve the best balance between accuracy and calculation time.

To be consistent with the MEF experiment, a 647 nm parallel laser field was used in the calculation. From our simulation results [Figures 2C, S3], we observed that the gold nanorod structure with a 15 nm minor axis and 45 nm major axis demonstrates the most notable enhancement of the electromagnetic field at its near field surrounding the surfaces of nanorod tips, with |E|_max_ > 5E_0_ for transverse excitation and |E|_max_ > 20E_0_ for longitudinal excitation, where |E|_max_ and E_0_ represent the maximum simulated and original electromagnetic field, respectively. Also, all nanorods showed much stronger MEF effects than spherical gold nanostructures. Most importantly, by performing UV–vis characterization on the library of gold nanorods and comparing their LSPR peak with the calculation results, we could clearly observe a similar trend as our simulation shows, with the 15 nm by 45 nm gold nanorods outperforming other structures in terms of the LSPR around the wavelength of 647 nm [Figure 2D]. Notably, our optical simulations and experimental characterization collectively demonstrated that gold nanorods with dimensions of 15 nm (minor axis) by 45 nm (major axis) generated the most pronounced LSPR-mediated MEF effect. These findings confirmed that these precisely dimensioned gold nanorods represent the optimal nanostructure for constructing the MEMB nanobiosensor, enabling superior sensitivity and signal stability for miRNA detection.

After identifying the optimal gold nanorod structure for maximizing the MEF effect, we hypothesized that this fluorescence enhancement could significantly improve the detection sensitivity of intracellular ncRNAs using MB-based biosensors. To validate this hypothesis, we performed ligand exchange reactions that replaced citrate capping molecules on the gold nanorod surfaces with thiol-modified (3′ end) molecular beacons designed to recognize miRNA-124 and labeled with Alexa Fluor 647 (5′ end) [Figure 3A]. After conjugation and purification, the MEMB was successfully constructed and characterized by the change of Zeta Potential [Figure S4] and the presence of phosphorus was confirmed by X-ray photoelectron spectroscopy (XPS) [Figures 3C, S5]. An artificial DNA mimicry of miRNA-124 was synthesized and tested as the target sequence in the solution test. To check if the MEF would lead to undesired background noise, we also tested the fluorescence at the “off” state of the MB by simply adding phosphate-buffered saline (PBS) into the MEMB solution. MEMB showed minimal signal in the fluorescence scan, confirming that the MEF does not enhance the “off” state background signal [Figure 3B]. However, once the target DNA sequence corresponding to miRNA-124 was added, fluorescence immediately illuminated. Additionally, the fluorescence was found to increase in response to an increase in miRNA-124 concentrations, indicating the potential of MEMB for quantitative measurement of miRNAs. Specifically, the detection limit of MEMB for miRNA-124 is around 10 pM, with a wide linear range from 10 pM to 10 μM [Figure 3E,F,G]. Lastly, to check the selectivity of the MEMB in the solution test, we compared the change in fluorescence signal in response to the target sequence and the mismatched sequence at an identical concentration of 100 nM. Minimal change was found in the group with the mismatched sequence added, confirming that MEMB is selective and capable of distinguishing single base pair mismatches [Figure 3D].

**Figure 3:**
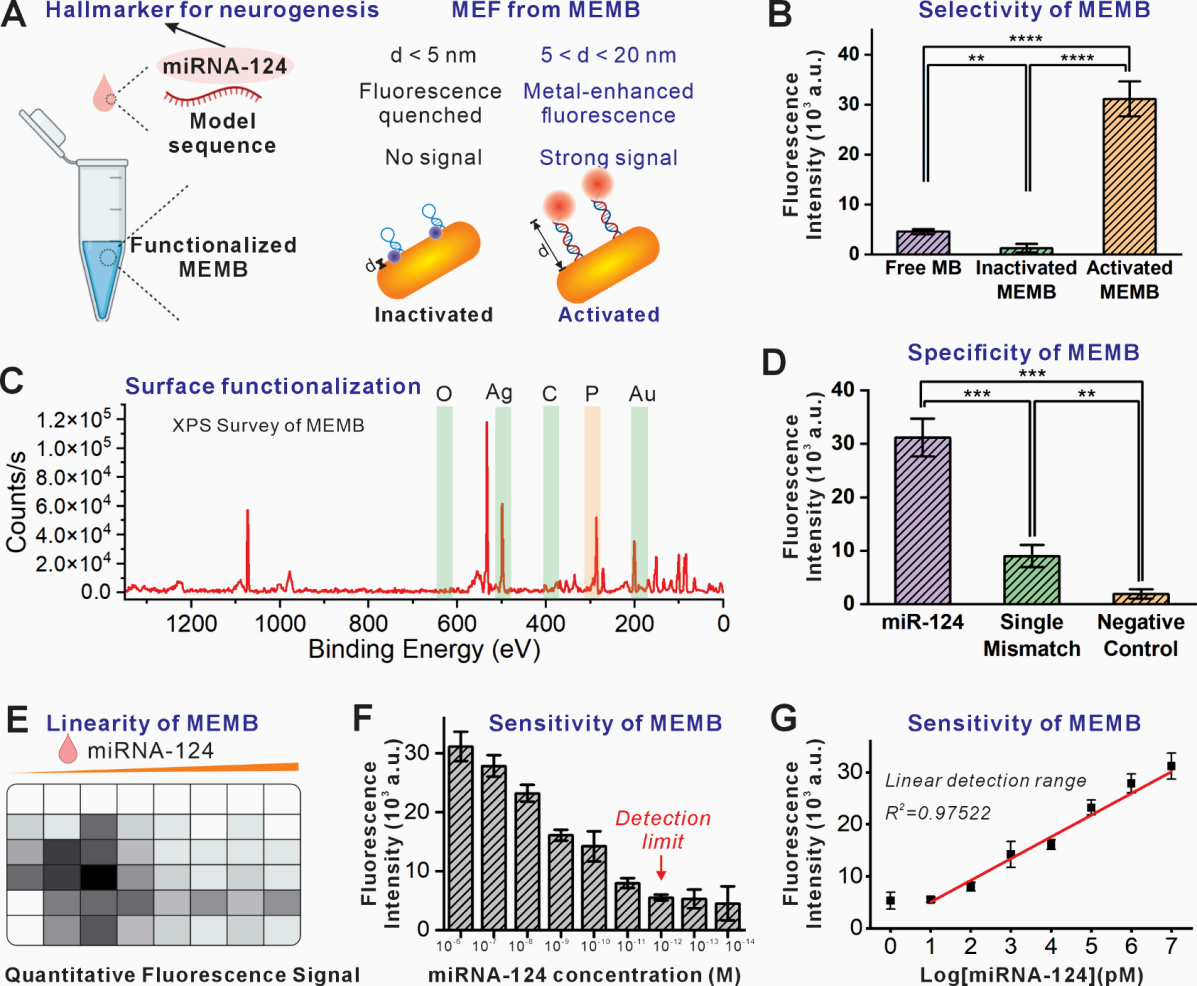
MEMB-based detection of miRNA-124 in solution. A) Illustration of MEF in the presence of the target sequence, d: distance between the dye and the surface of AuNR. B) Fluorescence turned on when the MEMB detected the presence of the miRNA-124 model sequence. C) XPS analysis demonstrated the successful conjugation of MEMB by identifying the presence of phosphorus, which only exists in the phosphate groups in the MB, but not in unconjugated AuNRs. D) High target selectivity demonstrated by the minimal fluorescence signal from the negative control (miRNA-67) and single-mismatch sequence. E,F) Concentration assay of miRNA-124. The detection limit of miRNA-124 using the MEMB was measured in a solution assay to be 10 pM. G) A linear detection range from 10 pM to 10 μM was established with *R* ^2^ = 0.97522. Statistical analysis by Student’s unpaired *t* test, *n* = 3. *p < 0.05, **p < 0.01, ***p < 0.001, ****p < 0.0001.

Building on MEMB’s exceptional sensitivity and selectivity demonstrated in solution-based assays, we proceeded to evaluate its capability for real-time monitoring of intracellular miRNA-124 dynamics during iPSC-NSC neuronal differentiation. Since miRNA-124 primarily functions within the intracellular environment during differentiation, we first assessed the cellular uptake efficiency of MEMB nanorods by iPSC-NSCs. Previous studies using cancer cell lines (e.g., HeLa) have revealed that optimal sizes of gold nanorods are between 40 and 50 nm,^70,71^ but such studies on iPSC-NSCs have been relatively lacking. We first tested the biocompatibility of MEMB at a wide concentration range of up to 200 μg/mL [Figure 4B]. Minimal changes in cell viability were observed at the highest MEMB concentration, demonstrating no cytotoxicity to iPSC-NSCs with MEMB concentrations up to 100 μg/mL. Then, cellular uptake of gold nanorods was studied at 4, 8, and 24 h after treating 2 mL of 50 μg/mL gold nanorods to 200k iPSC-NSCs seeded in a 6-well plate, separately [Figure S7]. The amount of cellular uptake was calculated by subtracting the amount of gold nanorods that remained in the solution, measured by extinction at 400 nm using a plate reader, from the total treated amount. From our results, iPSC-NSCs showed rapid uptake of gold nanorods within 4 h and continuous uptake from 4 to 8 h after treatment. Therefore, we have optimized and confirmed the sensitivity, selectivity, and feasibility of MEMB for intracellular detection of nucleic acids in iPSC-NSCs.

**Figure 4:**
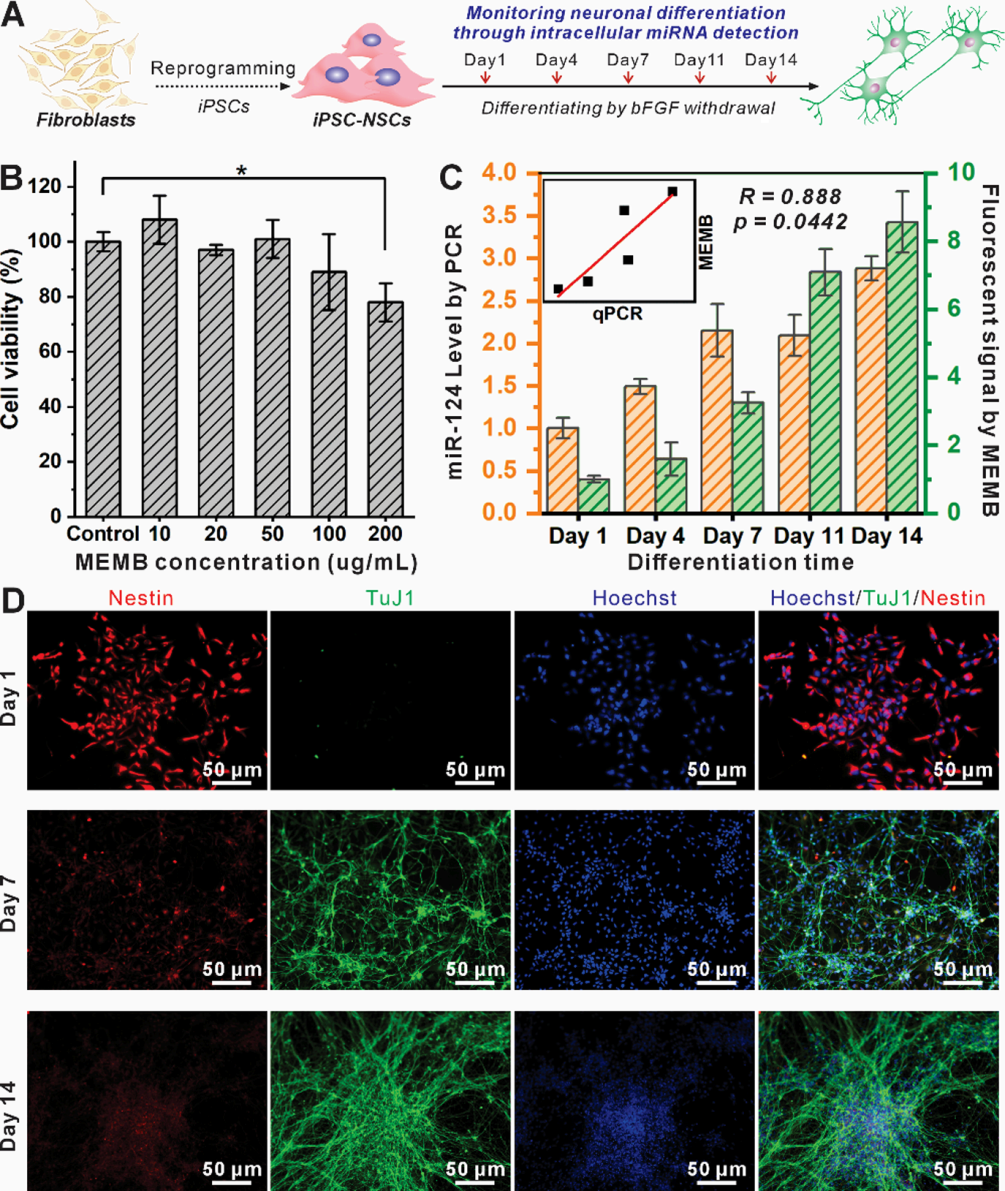
In vitro monitoring of iPSC-NSC differentiation using MEMB. A) Timeline of the two-week iPSC-NSC differentiation assay monitored using MEMB, qPCR, and immunocytochemistry. B) Cytotoxicity assay showing good biocompatibility of MEMB in a wide range of concentrations up to 100 μg/mL. C) miRNA-124 expression levels characterized by qPCR (orange) and by MEMB (green). Insert: Pearson correlation of qPCR and MEMB method measuring miRNA-124 expression level; Pearson correlation coefficient *R* = 0.888, *p* = 0.0442, suggesting a statistically significant correlation. D) Immunostaining of the neural stem cell marker Nestin (red) and neuron marker TuJ1 (green) at Day 1, Day 7, and Day 14 time points. Statistical analysis by Student’s unpaired *t* test, *n* = 3. *p < 0.05.

Next, we cultured iPSC-NSCs and induced their neuronal differentiation following standard procedures that we have established previously.^72^ The withdrawal of basic growth factor II (bFGF) initiates the differentiation of iPSC-NSC into neurons in a spontaneous manner.^73,74^ During the differentiation, cells typically show upregulated miRNA-124 that inhibits Rap2a, which activates AKT and the GSK3b pathway to induce neuronal differentiation and dendritic branching.^75,76^

To confirm if the removal of bFGF resulted in the upregulation of neuronal differentiation via miRNA-124, we performed a differentiation assay and analyzed cells with immunostaining and qRT-PCR studies, respectively. To monitor the entire differentiation process, we also performed time-dependent studies by sacrificing cells at various time points, including 1, 4, 7, 11, and 14 days post-bFGF withdrawal [Figure 4A]. We used TuJ1 as a representative neuronal marker and Nestin as a neural stem cell marker for immunocytochemistry characterization [Figure 4D]. As expected, both the cell morphology and protein expression change (upregulated TuJ1 and downregulated Nestin) confirmed the time-dependent differentiation of the iPSC-NSC after the bFGF removal.

After validating the differentiation assay, we then applied the MEMB nanobiosensor to monitor the neuronal differentiation of iPSC-NSC via the detection of miRNA-124 [Figure 4C]. Specifically, iPSC-NSCs were seeded and differentiated for 1, 4, 7, 11, and 14 days and treated with identical (10 μg/mL) concentrations of MEMB for 4 h. Afterward, fluorescence activation of MEMB inside differentiated iPSC-NSC was measured using a plate reader. By normalizing the fluorescence intensity from activated MEMB, we observed miRNA-124 levels with 1.5, 3.2, 7.1, and 8.6-fold increases after being seeded for differentiation for 4, 7, 11, and 14 days, directly correlating to the differentiation states of the iPSC-NSCs. To confirm this, we also performed qRT-PCR analysis to calculate the miRNA-124 levels. Although the absolute copy number and concentrations of miRNA-124 measured in qRT-PCR are different from our MEMB test, their fold change across different time points shows a similar trend, further validating the MEMB for monitoring neuronal differentiation via the detection of miRNA-124. Notably, these analyses were performed in solution without sacrificing cell viability, showcasing advantages over conventional monitoring approaches.

Real-time monitoring of the dynamic intracellular distribution and expression levels of ncRNAs is essential for advancing iPSC technologies from the laboratory into clinical settings. However, existing methods often lack the sensitivity, selectivity, and noninvasive features required for effective intracellular ncRNA monitoring. Addressing these limitations, we have developed a highly sensitive, selective, and nondestructive MEMB nanobiosensor specifically designed to detect intracellular miRNA-124, a critical biomarker associated with neuronal differentiation. To achieve optimal MEF performance, we systematically employed FDTD simulations along with comprehensive experimental screening to identify and validate AuNRs with superior LSPR properties.

Importantly, our MEMB platform technology effectively amplified the fluorescence intensity of the MB conjugated with Alexa 647 dye by up to 30-fold, achieving picomolar-level sensitivity for miRNA-124 detection. Additionally, MEMB demonstrated high selectivity, successfully differentiating single-base-pair mismatches, thereby highlighting its precision. Crucially, this nanobiosensor was efficiently internalized by hiPSC-NSCs and facilitated the real-time, noninvasive monitoring of miRNA-124 dynamics throughout a two-week neuronal differentiation process. Unlike traditional methods such as immunostaining or qRT-PCR, MEMB does not necessitate fixation or cell harvesting, thus preserving cell viability and enabling longitudinal analysis within the same batch of cells. Moreover, MEMB allows for the visualization of spatial variations of miRNA expression across individual cells. In parallel, the two-step surface modification further enhanced the stability and repeatability of the MEMB biosensor with low signal variation. Consequently, this nanobiosensor presents significant advantages over existing methodologies, offering a powerful new approach for studying the spatiotemporal regulation of ncRNAs during iPSC differentiation, ultimately contributing to improved models and therapies for CNS diseases and injuries.

Moving forward, advancing the MEMB nanobiosensor toward multiplexed detection of diverse biomolecular targets, such as specific ncRNAs, proteins, and signaling molecules, represents a critical advancement, as this capability would provide comprehensive insights into the complex molecular and genetic networks governing iPSC differentiation. Deciphering the intricate regulatory mechanisms driving iPSC neurogenesis would be challenging without such multiplexed detection systems. Furthermore, while our current study demonstrates MEMB’s capacity to reveal spatial distributions of miRNAs across cell populations, integrating this technology with advanced single-cell sorting platforms and genomic analysis methods would significantly enhance our understanding of cellular heterogeneity during differentiation. Additionally, as the conductivity of material could affect the neural differentiation of stem cells, it would be interesting to investigate whether the conductivity of gold nanorods would induce cell membrane polarization and iPSC differentiation. Finally, although our MEMB platform enables real-time, noninvasive monitoring of general neuronal differentiation through miRNA-124 detection, expanding this approach to identify and distinguish specific neuronal subtypes represents an important future objective. Achieving subtype-specific monitoring would substantially broaden MEMB’s applications, enabling more precise characterization of iPSC-derived neural populations and unlocking new possibilities for clinical translation of cell-based therapies for central nervous system disorders.

## Supplementary Material



## Notes

Details about the methods, design of molecular beacon and single-strand DNA targets, antibodies used in immunocytochemistry experiments, and supplementary figures on gold nanorods synthesized with various sizes and aspect ratios, UV–vis spectra of nanorods, FDTD simulation of electromagnetic field enhancement and scattering cross-section of AuNRs, Zeta potential of AuNRs before and after ligand exchange and molecular beacon conjugation, successful conjugation of MEMB confirmed by XPS, cytotoxicity assay of surface-functionalized AuNRs, and cellular uptake of gold nanorods by iPSC-NSC cells (PDF)
