# Supplementary material for: Real-Time Intracellular Monitoring of miRNA Dynamics during Induced Pluripotent Stem Cell Neuronal Differentiation via Plasmon-Enhanced Nanobiosensing
Source: Nano Lett. 2025 Jun 10;25(26):10402–11. doi: 10.1021/acs.nanolett.5c01840 (PMC12333427; doi:10.1021/acs.nanolett.5c01840)
Supplement: Supplementary file 1 [file nl5c01840_si_001.pdf]

SUPPORTING INFORMATION

Real-time Intracellular Monitoring of miRNA Dynamics During iPSC Neuronal Differentiation via Plasmon-Enhanced Nanobiosensing

Yannan Hou[1], Meizi Chen[1], Letao Yang[1,2], and Ki-Bum Lee[1,]**

[1] Department of Chemistry and Chemical Biology, Rutgers, The State University of New Jersey, Piscataway, NJ 08854, USA

[2] Shanghai Tongji Hospital, Key Laboratory of Spine and Spinal Cord Injury Repair and Regeneration, Ministry of Education, Frontier Science Center for Stem Cell Research, School of Life Sciences and Technology, Tongji University, Shanghai 200092, China.

***CORRESPONDING AUTHORS:**
Prof. Ki-Bum Lee
Department of Chemistry and Chemical Biology
Rutgers, The State University of New Jersey, 123 Bevier Road, Piscataway, NJ 08854, USA
Phone: +1-732-445-2081; Fax: +1-732-445-5312
E-mail: kblee@rutgers.edu
Website: https://kblee.rutgers.edu/

Prof. Letao Yang
Shanghai Tongji Hospital, School of Life Science and Technologies, Tongji University, Shanghai 200065, China
E-mail: yangletao@tongji.edu.cn

KEYWORDS: Intracellular miRNA Detection; Real-time Monitoring; Gold Nanorods; Non-destructive biosensor; Monitoring iPSC differentiation; Metal-Enhanced Fluorescence (MEF); Neuronal Differentiation

TABLE OF CONTENTS

A. METHODS

B. SUPPLEMENTARY TABLES AND FIGURES

TABLE S1. Design of molecular beacon and single-strand DNA targets.

TABLE S2. Antibodies used in immunocytochemistry experiments.

FIGURE S1. Gold nanorods synthesized with various sizes and aspect ratios.

FIGURE S2. UV-Vis spectra of synthesized gold nanorods.

FIGURE S3. FDTD simulation of electromagnetic field enhancement and scattering cross-section of AuNRs with various aspect ratios.

FIGURE S4. Zeta potential of AuNRs before and after ligand exchange and molecular beacon conjugation.

FIGURE S5. Successful conjugation of MEMB confirmed by XPS.

FIGURE S6. Cytotoxicity assay of surface-functionalized AuNRs.

FIGURE S7. Cellular uptake of gold nanorods after 4, 8, and 24 hours of incubation with iPSC-NSC.

METHODS

Synthesis of Gold Nanorods

Gold nanorods (AuNRs) with an average diameter of 15 nm and a length of 45 nm were synthesized using a seed-mediated growth method in the presence of cetyltrimethylammonium bromide (CTAB) as a surfactant, following a modified protocol adapted from Murphy et al. **i) Preparation of Gold Seed Solution.** A gold seed solution was prepared by reducing gold ions in the presence of CTAB. First, 9.75 mL of 0.1 M CTAB was added to 0.25 mL of 0.01 M $HAuCl_4 \cdot 3H_2O$ in a 50 mL round-bottom flask pre-cleaned with aqua regia. Next, 0.60 mL of freshly made ice-cold 0.01 M $NaBH_4$ solution was rapidly added under vigorous stirring, forming the seed solution with a light brownish-yellow color. The solution was stirred for an additional 2 minutes and then left undisturbed in a 27 °C water bath for 2 hours to ensure complete stabilization of the gold seeds. **ii) Growth of Gold Nanorods.** A growth solution was prepared by combining 5 mL of 0.2 M CTAB, 5 mL of 1.0 mM $HAuCl_4$, and 0.25 mL of 4.0 mM $AgNO_3$ in a 20 mL glass vial. This mixture was then added to 70 μL of 0.0788 M ascorbic acid, resulting in a colorless solution under gentle stirring. Subsequently, 12 μL of the gold seed solution was added to the growth solution, and the mixture was gently stirred for 30 seconds. The reaction vial was then left undisturbed at 27 °C for 12 hours to allow the growth of gold nanorods. **iii) Purification of Gold Nanorods.** The synthesized gold nanorods were purified by centrifugation at 10,000 rpm for 10 minutes and washed with deionized water twice to remove excess gold seeds and gold nanoparticles smaller than the desired gold nanorods. High monodispersity of the purified gold nanorods was confirmed by TEM (Figure S1) and UV-Vis spectra (Figure S2). For a typical 40 mL batch of synthesized AuNRs, the pellet was redispersed in 2 mL deionized water to make a 20X concentration stock AuNR solution for temporary storage before ligand exchange. **iv) Characterization.** The morphology and dimensions of the gold nanorods were characterized using transmission electron microscopy (TEM). The average diameter and length of the nanorods were determined to be 15 nm and 45 nm, respectively, consistent with the targeted dimensions. UV-Vis spectroscopy was employed to confirm the presence of longitudinal and transverse plasmon resonance peaks, which are characteristic of gold nanorods. Nanorod concentration was estimated by the absorption at 400 nm.

Finite-Difference Time-Domain (FDTD) simulation

FDTD simulations were performed to investigate the enhancement of the electromagnetic field and scattering cross-section of AuNRs and gold nanoparticles (30 nm diameter) using Lumerical FDTD Solutions. AuNRs were modeled as cylindrical rods with diameters of 15 nm, aspect ratios of 1, 2, 3, 4, and 5, separately, and were immersed in water (n = 1.33). The dielectric function of gold was defined using experimental data from Johnson and Christy. A uniform mesh with a fine resolution of 0.5 nm near the nanorod surface was applied, and perfectly matched layer (PML) boundary conditions were used to prevent reflections. A plane wave source, polarized along the longitudinal axis of the nanorod, illuminated the structure, and the wavelength was swept from 400 to 1200 nm to capture the plasmonic response. Frequency-domain field monitors recorded the electric field distribution, and the field enhancement factor was calculated as the ratio of the local electric field intensity to the incident field intensity. A far-field monitor computed the scattering cross-section by integrating the scattered power over a solid angle and normalizing it to the incident power. It produced a wavelength-dependent scattering spectrum with peaks corresponding to transverse and longitudinal plasmon resonances.

Surface Ligand Exchange of Gold Nanorods

The surface ligand exchange of AuNRs synthesized with CTAB to poly(sodium 4-styrenesulfonate) (PSS) and subsequently to citrate was performed following a modified protocol based on methods described by Wei et al. (Langmuir 2014, 30, 46, 13727–13730). Typically, 2 mL of purified CTAB-coated AuNRs (20X) were added into 8 mL of 0.15 wt% Na-PSS in a drop-by-drop manner under gentle stirring at room temperature. The mixture was then incubated at room temperature for 2 hours under gentle stirring to ensure complete ligand exchange, followed by centrifuging at 7,500 × g for 30 minutes. The supernatant was decanted until 0.2 mL of the retentate remained. AuNRs were redispersed in 4 mL of 0.7 wt% Na-PSS, incubated at room temperature for 2 hours under gentle stirring, and centrifuged at 7,500 × g for 30 minutes. The supernatant was decanted until 0.2 mL of the retentate remained. The PSS-coated AuNRs were diluted with DI water to a final volume of 1 mL, and were then added to 9 mL of 5 mM sodium citrate drop-by-drop under gentle stirring at room temperature. The mixture was then incubated at room temperature for 2 hours under gentle stirring to ensure complete ligand exchange. Citrate-coated AuNRs were centrifuged at 7,500 × g for 30 minutes and redispersed in 5 mM sodium citrate. Purified citrate-AuNRs can be stored at room temperature for at least two months.

Conjugating Molecular Beacons onto Citrate-Stabilized Gold Nanorods

First, the thiol group on the single-strand DNA molecular beacon is deprotected by reducing the disulfide bond using tris(2-carboxyethyl) phosphine (TCEP). The molecular beacon is dissolved in nuclease-free water to a concentration of 100 μM, and TCEP is added in excess at a 100:1 molar ratio relative to the DNA. The mixture is incubated at room temperature for 2 hours to ensure complete reduction of the thiol group. The molecular beacon is then mixed with citrate-stabilized AuNRs at a molar ratio of 100:1 (DNA: AuNR) and incubated for 2 hours to allow the thiolated DNA to bind to the AuNR surface via Au-S bonds. To stabilize the DNA-AuNR conjugates, a salt-aging step is performed by gradually increasing the ionic strength of the solution. Small aliquots of 1 M NaCl are added in a drop-by-drop manner every 15 minutes until the final NaCl concentration reaches 0.1 M, followed by incubation at room temperature for 24 hours. This step screens the electrostatic repulsion between the negatively charged DNA strands, enabling higher DNA loading on the AuNR surface. After conjugation, the DNA-AuNR complexes are purified by centrifugation at 10,000 × g for 10 minutes to remove unbound DNA and excess salt, with the pellet redispersed in nuclease-free water. The process is repeated twice to ensure complete purification.

X-ray photoemission spectroscopy (XPS)

Silicon substrates (1 cm x 1 cm) cut from prime-grade silicon wafers were used for XPS sample preparation. The substrates were thoroughly cleaned by sonicating them for 30 minutes in a 1% Triton X-100 solution, followed by sonicating in DI water for 20 minutes and ethanol for 20 minutes. They were then dried in a vacuum desiccator. Citrate-stabilized AuNR and DNA-conjugated MEMB solutions were drop-cast onto the cleaned silicon substrates separately with a minimum volume of 10 μL and dried overnight under vacuum at room temperature. XPS measurements were performed on a Thermo K-alpha instrument using a monochromatized Al Kα photon source with a 400 μm x-ray spot and photon energy of 1486.7 eV. Survey spectra and core-level spectra were recorded in constant analyzer energy mode with pass energies of 200 eV and 50 eV, and energy step sizes of 1.00 eV and 0.100 eV, respectively. Core-level spectra were charge-corrected and referenced to the binding energy of the C–C bond at 284.8 eV. Data analysis, including element identification and peak fitting, was conducted using the Avantage Data System.

Cell culture and differentiation

Induced pluripotent stem cell-derived neural progenitor cells (iPSC-NSCs) were cultured in neural progenitor cell (NSC) maintenance medium, consisting of a mixture of neural basal medium (Gibco) and DMEM/F12 (Gibco) (50:50 ratio) supplemented with 1× N-2 supplement (Gibco), 1× B-27 supplement (Gibco), 20 ng/mL recombinant human bFGF (fibroblast growth factor-basic, PeproTech). Culture plates were coated with a suitable extracellular matrix to promote cell adhesion and growth. Briefly, 6-well plates were coated with 1 mL per well of a 1:100 dilution of Matrigel (Corning) in DMEM/F-12 medium (Gibco) and incubated at 37°C for at least 1 hour. Before cell seeding, the coating solution was aspirated, and the plates were rinsed once with DMEM/F-12 medium. Cells were seeded at a density of 1 × 10^5 cells per cm^2 onto the Matrigel-coated plates and were maintained in NSC maintenance medium at 37°C in a humidified incubator with 5% CO2. The medium was replaced every other day to ensure a consistent and optimal nutrient supply. To differentiate cells, iPSC-NSCs were seeded in NSC maintenance medium. After 1 day of cultivation to promote cell attachment and spreading, the cell medium was replaced by NSC differentiation medium consisting of a mixture of neural basal medium and DMEM/F12 (50:50 ratio) supplemented with 1× N-2 supplement and 1× B-27 supplement to stop proliferation and induce neuronal differentiation. The medium was changed with fresh differentiation media every 2 to 3 days during the differentiation.

Immunocytochemistry

Immunocytochemistry was conducted to confirm the neuronal differentiation of iPSC-NSCs after withdrawing bFGF. Cells were fixed with a 4% formaldehyde solution (Sigma-Aldrich) at room temperature for 10 minutes and washed with PBS. Cells were then permeabilized with 0.3% Triton X-100 in PBS, and non-specific binding was blocked with 5% normal goat serum (Gibco) at room temperature for 1 hour. Samples were incubated with primary antibody solutions diluted in PBS with 1% bovine serum albumin (BSA) at 4°C overnight, washed with PBS three times, and then incubated with appropriate fluorophore-labeled secondary antibodies diluted in PBS with 1% BSA at room temperature for 1 hour in the dark. Cells were washed to eliminate non-specific binding and incubated with DNA labeling dye Hoechst 33342 (Thermo Scientific) before mounting on microscope slides using Fluoromount aqueous mounting medium (Sigma-Aldrich). All fluorescence images were taken with a Nikon microscope.

Gene expression analysis by RT-qPCR

Messenger RNA qPCR. Undifferentiated iPSC-NSCs and cells differentiated for 1, 4, 7, and 11 days were lysed using TRIzol (Invitrogen). Total RNA extraction, precipitation, and purification were carried out following vendor protocol (TRIzol Reagent User Guide Pub. No. MAN0001271 C.0). RNA concentrations and A260/280 ratios were measured using a Nanodrop Lite Spectrophotometer (Thermo Scientific). Reverse transcription was carried out using a thermocycler (Eppendorf) with AccuPower CycleScript RT PreMix (BIONEER). Quantitative PCR was performed using the StepOnePlus Real-Time PCR System (Applied Biosystems) with SYBR Green Universal Master Mix (Thermo Scientific). GAPDH was used as an endogenous control. Relative mRNA expression levels were calculated using the $\Delta\Delta Ct$ method and normalized to gene expression of undifferentiated iPSC-NSCs.

Micro RNA qPCR. The quantification of miRNA-124 was performed using the miRCURY LNA miRNA PCR System (QIAGEN) according to the manufacturer's protocol. Total RNA was extracted from samples using the miRNeasy Mini Kit (QIAGEN), and RNA concentration and purity were assessed using a NanoDrop. cDNA synthesis was carried out using the miRCURY LNA RT Kit, where 10 ng of total RNA was reverse-transcribed

in a 10 µL reaction volume with a universal poly(A) tailing and cDNA synthesis step. The resulting cDNA was diluted and used as a template for qPCR with miRNA-specific LNA-enhanced primers for miRNA-124 and a miRCURY LNA SYBR Green PCR Kit. Reactions were performed in triplicate using the StepOnePlus Real-Time PCR System (Applied Biosystems) under the following cycling conditions: 95°C for 2 minutes, followed by 40 cycles of 95°C for 10 seconds and 60°C for 1 minute. A melt curve analysis was conducted to confirm the specificity of the amplification. Relative quantification of miRNA-124 expression was determined using the $2^{(-\Delta\Delta Ct)}$ method, using miRNA-103-3p as an endogenous control.

SUPPLEMENTARY TABLES AND FIGURES

Table S1. Design of molecular beacon and single-strand DNA targets (positive, single mismatch, and negative control).

	**DNA Sequence**
Molecular beacon	/5Alex647N/TTG GCA TTC ACC GCG TGC CTT AGC CAA AAA AAA AAA A /3ThioMC3-D/
Positive control (miR-124)	5' TTA AGG CAC GCG GTG AAT GCC A 3'
Single mismatch	5' TTA AGG CAC G**T**G GTG AAT GCC A 3'
Negative control (miR-67)	5' TCA CAA CCT CCT AGA AAG AGT AGA 3'

Table S2. Antibodies used in immunocytochemistry experiments.

	Primary Antibody and Dye			Secondary Antibody		
**Target**	**Antibody**	**Vendor, Catalog #**	**Dilution**	**Antibody**	**Vendor, Catalog #**	**Dilution**
TuJ1	Mouse anti-TUJ1/b tubulin	Invitrogen, 480011	1:200	Alexa Fluor 488 Goat anti-mouse	Invitrogen, A11034	1:200
Nestin	Rabbit anti-Nestin	Thermo Fisher, PA511887	1:300	Alexa Fluor 546 Goat anti-rabbit	Invitrogen, A11030	1:200
Nucleus	Hoechst 33342	Thermo Scientific, 62249	1:1,000	-	-	-

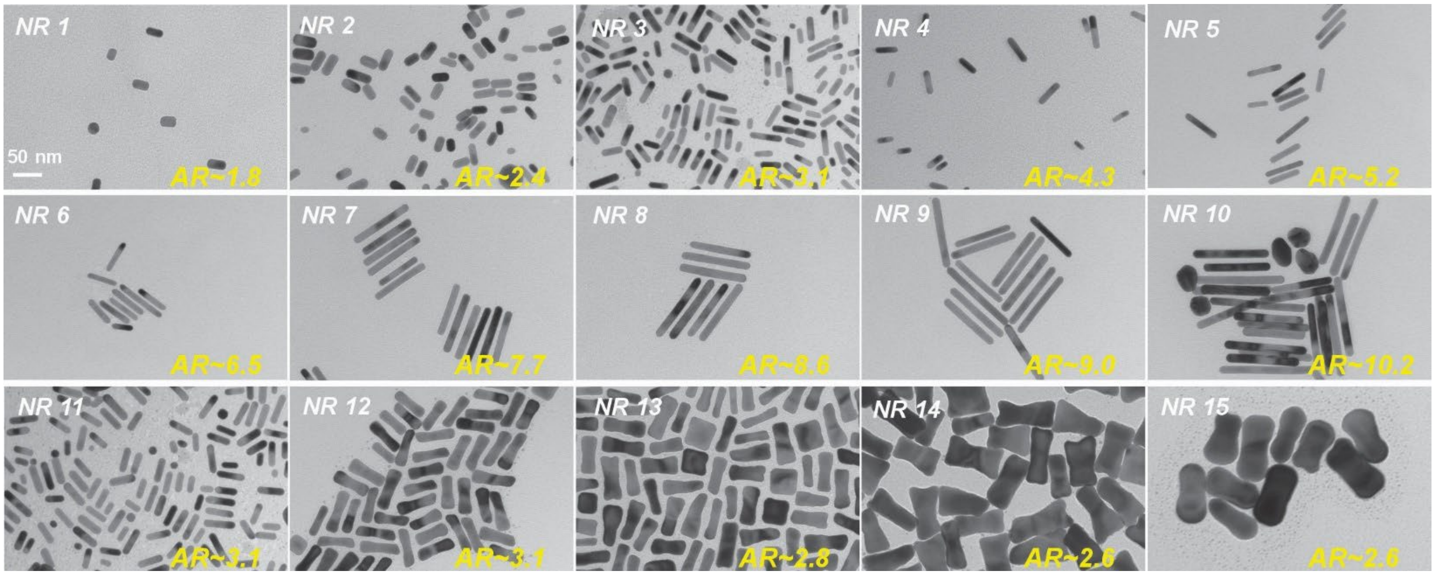

Figure S1. Gold nanorods were synthesized with various sizes and aspect ratios. Nanorod morphologies and aspect ratios (ARs) were characterized using TEM.

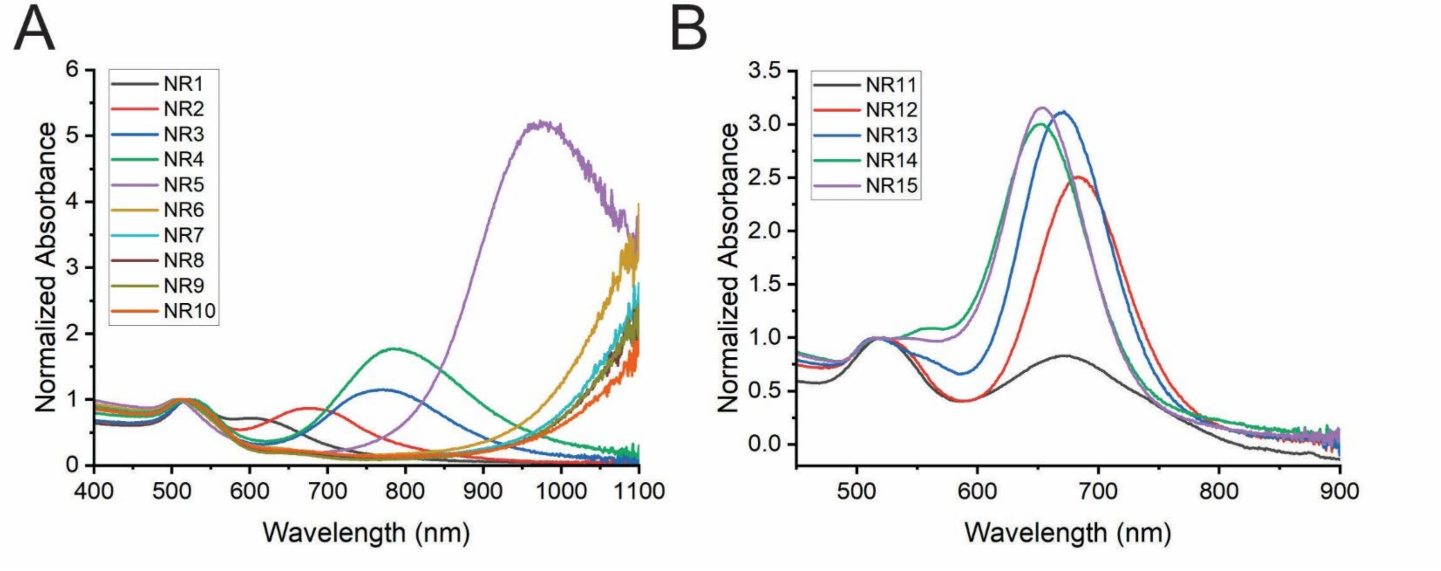

Figure S2. UV-Vis spectra of nanorods showing a universal transverse plasmon peak around 520 nm and a longitudinal plasmon peak corresponding to the aspect ratio. A) Gold nanorods with an aspect ratio ranging from around 1 to over 10; B2) gold nanorods with an aspect ratio of around 3 with various sizes.

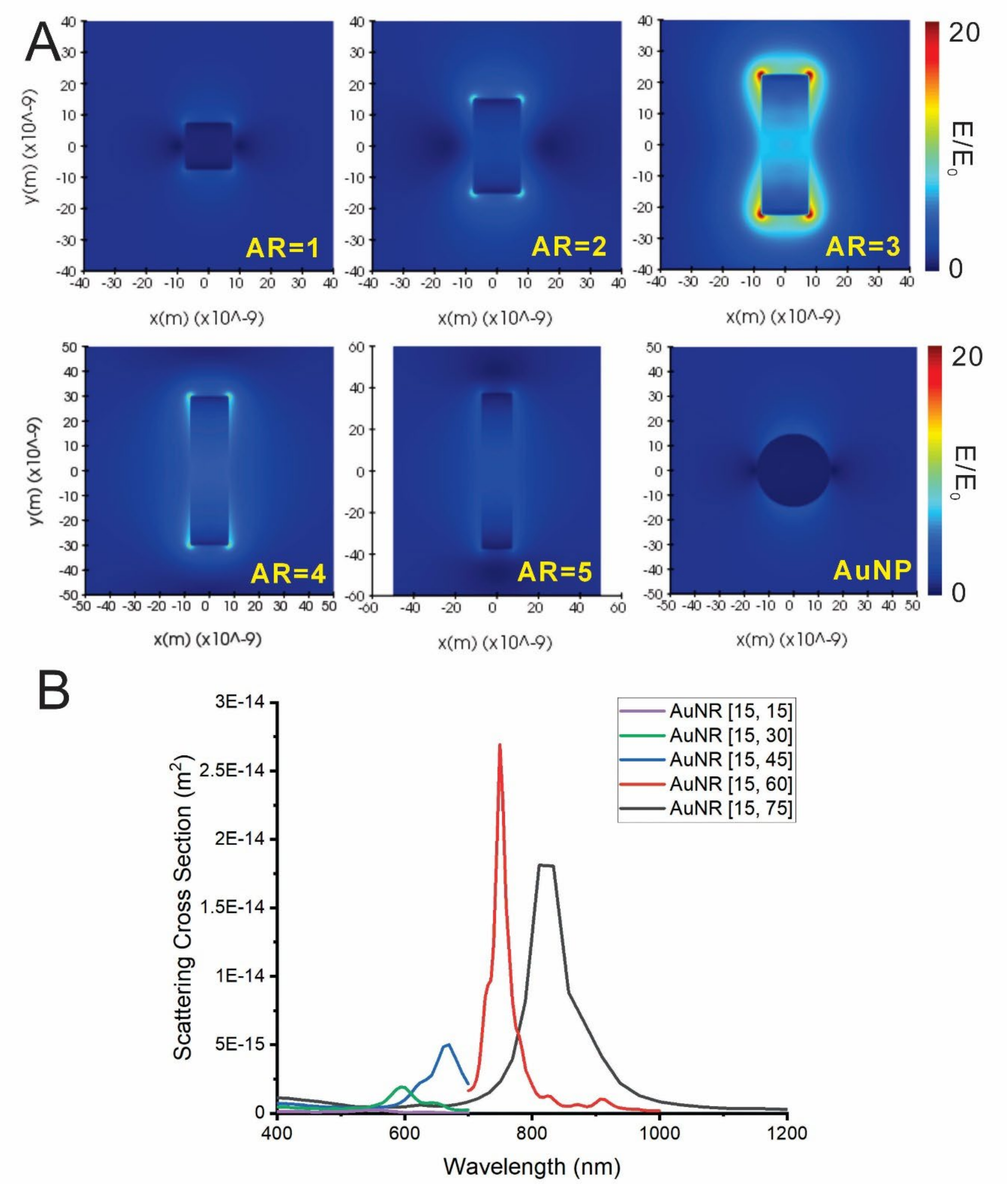

Figure S3. FDTD simulation of electromagnetic field enhancement and scattering cross-section of AuNRs with various aspect ratios. A) Electromagnetic field enhancement of AuNRs and Au nanoparticles (d = 15 nm) at 647 nm excitation; B) Scattering cross-section of AuNRs with 5 different ARs.

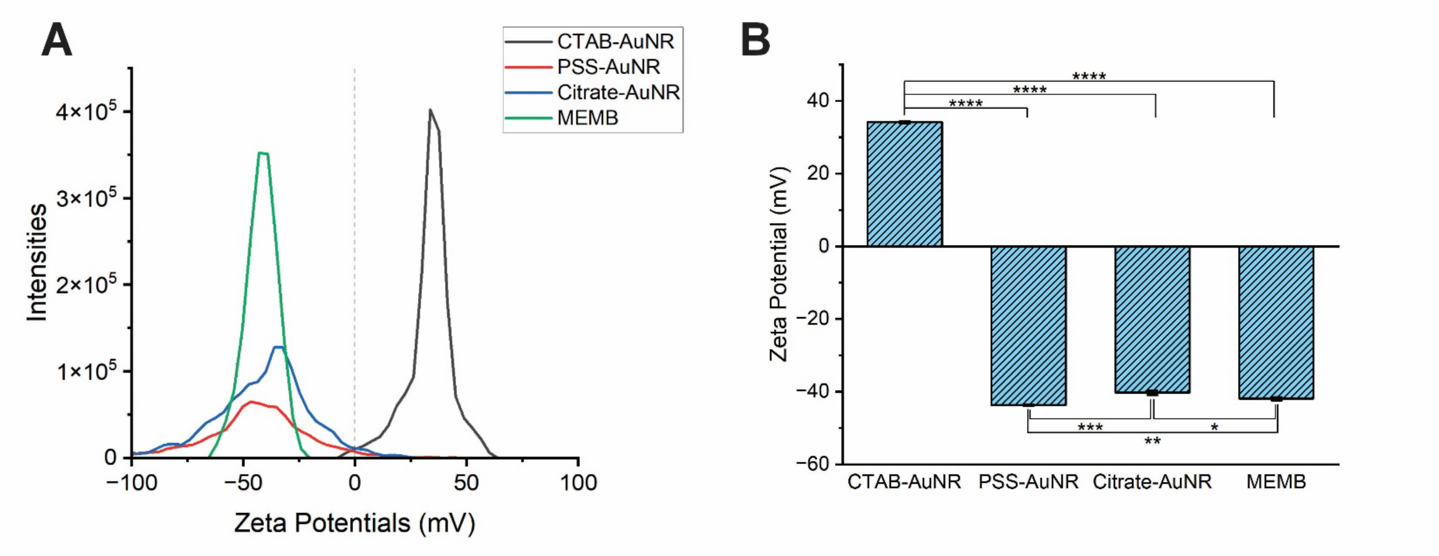

Figure S4. Zeta potential of AuNRs before and after ligand exchange and molecular beacon conjugation. Data are shown as mean ± stdev. n = 3 experimental replicates. Statistical analysis by student's unpaired t-test. *$p < 0.05$, **$p < 0.01$, ***$p < 0.001$, ****$p < 0.0001$.

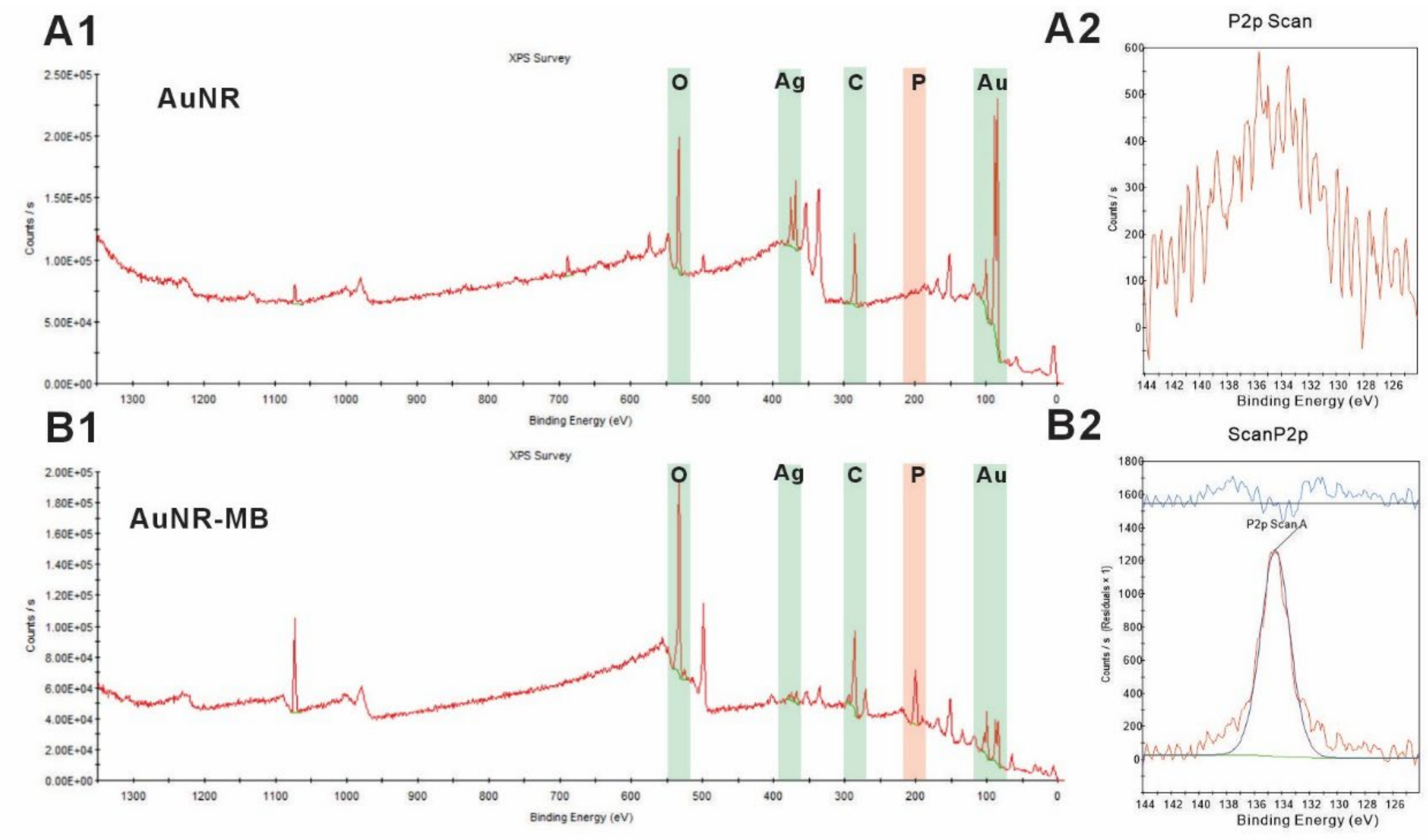

Figure S5. Successful conjugation of MEMB confirmed by XPS. A) XPS survey spectra (A1) and a core-level scan of phosphate (A2) proved the absence of phosphorus on the AuNR surface before nucleotide conjugation; B) XPS survey spectra (B1) and a core-level scan of phosphorus (B2) showed the existence of phosphorus (phosphate group in the nucleotide) on the molecular beacon-conjugated AuNR, confirming the successful assembly of MEMB.

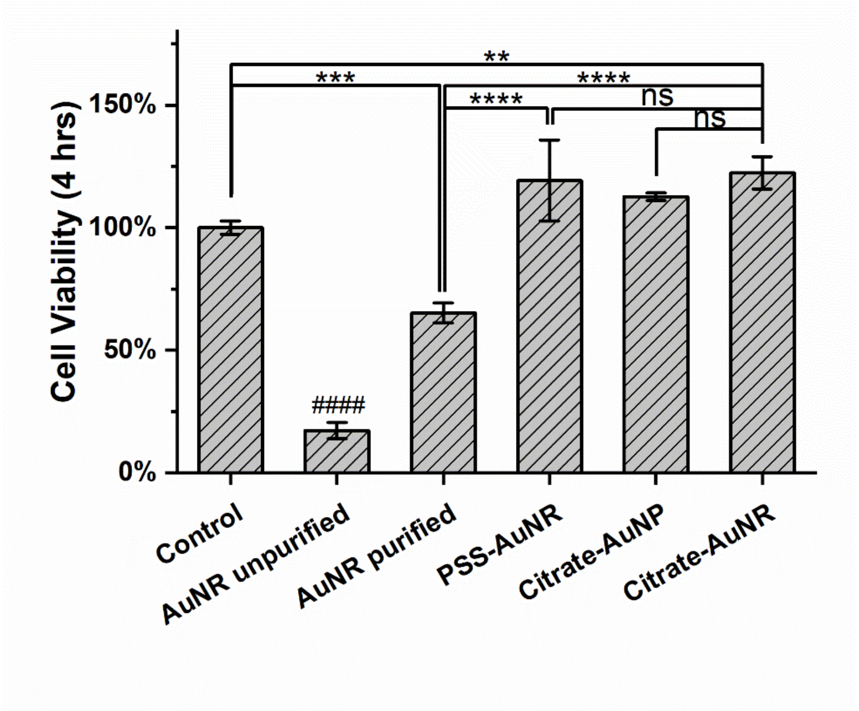

Figure S6. Cytotoxicity assay of surface-functionalized AuNRs. Relative viabilities of iPSC-NSCs showing significantly enhanced biocompatibility of gold nanorods after ligand exchange. Data are shown as mean ± stdev. n = 3 experimental replicates. Statistical analysis by student's unpaired t-test. ns = not significant, *$p < 0.05$, **$p < 0.01$, ***$p < 0.001$, ****$p < 0.0001$, $^{\#\#\#\#}p < 0.0001$ compared to all other groups.

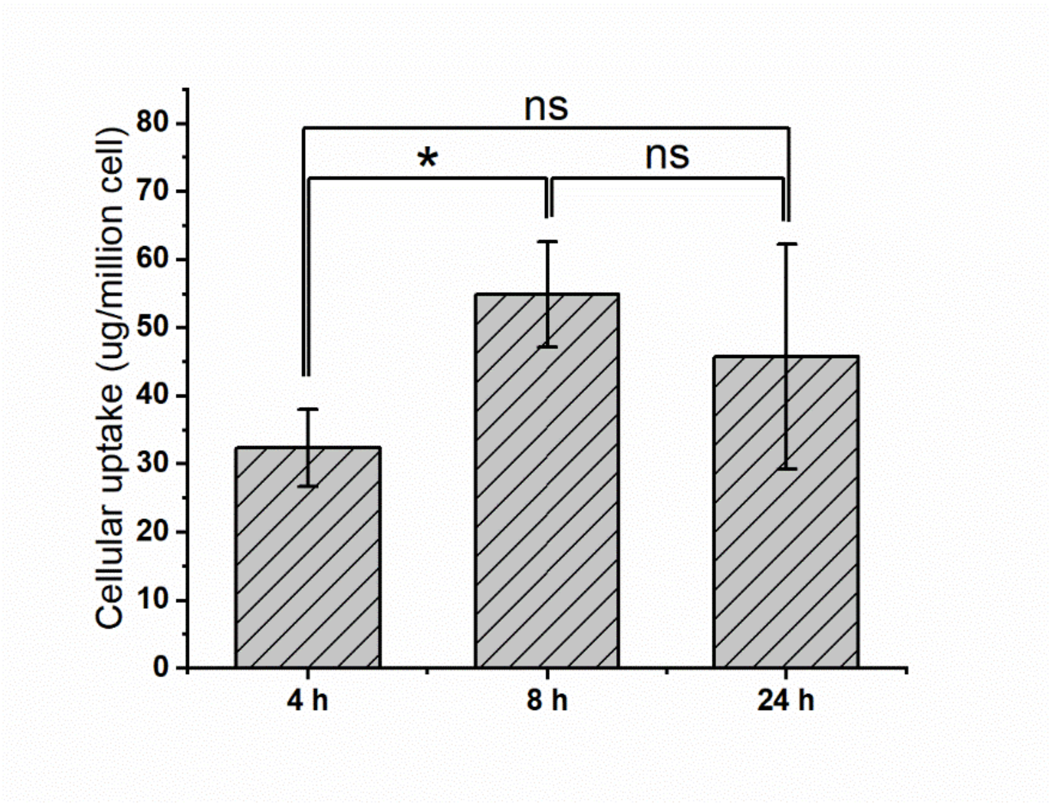

Figure S7. Cellular uptake of gold nanorods after 4, 8, and 24 hours of incubation with iPSC-NSC cells. The amount of AuNRs uptake by iPSC-NSCs was calculated from the total amount of treatment subtracted by the amount of AuNRs left in the cell media after treatment, measured by absorption at 400 nm using a plate reader. Statistical analysis by student's unpaired t-test, $n = 3$. ns = not significant, *$p < 0.05$.
